# Unprecedented increase of West Nile virus neuroinvasive disease, Spain, summer 2020

**DOI:** 10.2807/1560-7917.ES.2021.26.19.2002010

**Published:** 2021-05-13

**Authors:** Lucía García San Miguel Rodríguez-Alarcón, Beatriz Fernández-Martínez, María José Sierra Moros, Ana Vázquez, Paula Julián Pachés, Elena García Villacieros, María Belén Gómez Martín, Jordi Figuerola Borras, Nicola Lorusso, Julian Mauro Ramos Aceitero, Elena Moro, Aránzazu de Celis, Salvador Oyonarte, Beatriz Mahillo, Luis José Romero González, María Paz Sánchez-Seco, Berta Suárez Rodríguez, Ulises Ameyugo Catalán, Santiago Ruiz Contreras, Mayte Pérez-Olmeda, , Fernando Simón Soria

**Affiliations:** 1 Ministry of Health, Coordinating Center of Health Alerts and Emergencies, Madrid, Spain; 2 National Center for Epidemiology, Instituto de Salud Carlos III, Ministry of Science and Innovation, , Madrid, Spain; 3 CIBER Epidemiology and Public Health, Madrid, Spain; 4 National Microbiology Center, Instituto de Salud Carlos III, Ministry of Science and Innovation; 5 Consorcio Hospital General de Valencia, Valencia, Spain; 6 Ministerio de Agricultura, Pesca y Alimentación, Dirección General de Sanidad de la Producción Agraria, Madrid, Spain; 7 Consejo Superior de Investigaciones Científicas, Estación biológica de Doñana, Seville, Spain; 8 Junta de Andalucía, Servicio de Vigilancia y Salud laboral Seville, Spain; 9 Junta de Extremadura, Subdireccion de Epidemiologia, Mérida-Badajoz, Spain; 10 Ministry of Health, Scientific Committee on Transfusion Safety, Madrid, Spain; 11 Organización Nacional de Trasplantes Madrid, Spain; 12 Junta de Andalucía. Consejería de Salud y Familias. Dirección General de Salud Pública y Ordenación Farmacéutica. Subdirección de Protección de la Salud, Seville, Spain; 13 Diputación de Hueva. Servicio de Control de Mosquitos, Huelva, Spain

**Keywords:** Spain, vector-borne infections, viral infections, West Nile fever, West Nile virus, WNV, surveillance, epidemiology

## Abstract

Cases of West Nile neuroinvasive disease (WNND) in Spain increased in summer 2020. Here we report on this increase and the local, regional and national public health measures taken in response. We analysed data from regional surveillance networks and the National Epidemiological Surveillance Network, both for human and animal West Nile virus (WNV) infection. During the 2020 season, a total of 77 human cases of WNV infection (median age 65 years; 60% males) were detected in the south-west of Spain; 72 (94%) of these cases developed WNND, presenting as meningoencephalitis, seven of which were fatal. In the previous two decades, only six human cases of WNND were detected in Spain. Reduced activities for vector control this season, together with other factors, might have contributed to the massive increase. Public health measures including vector control, campaigns to raise awareness among physicians and the general population, and interventions to ensure the safety of donations of blood products, organs, cells and tissues were effective to reduce transmission. Going forward, maintenance of vector control activities and an update of the vector-borne diseases response plan in Spain is needed.

## Background

West Nile virus (WNV) is a zoonotic pathogen transmitted from birds to mammals, in particular humans and horses, by *Culex* mosquitoes. WNV lineages 1 and 2 have been described to affect humans, in whom the majority of infections are asymptomatic; 20–40% of cases develop West Nile fever (WNF), which presents as influenza-like symptoms after an incubation period of 3–15 days. West Nile neuroinvasive disease (WNND)—which can present as meningitis, encephalitis or acute flaccid paralysis—develops in less than 1% of cases. WNND has a highly variable clinical course, but is often associated with considerable long-term morbidity. Approximately two-thirds of those with paralysis continue to experience significant weakness in the affected limbs [1]. Treatment is supportive, and after the acute phase recovery long-lasting immunity is conferred. Nevertheless, sequelae are frequent, especially after WNND, and are also possible after WNF. The most frequent physical, psychological and functional sequelae are, respectively, muscle weakness, memory loss and difficulty doing activities of daily living. The WNND fatality rate is around 10%. Several risk factors have been identified for both WNND and death, with advanced age, male sex and underlying medical conditions being the most common ones. There is no WNV vaccine for humans [2].

The first WNV infection cases in Europe were detected in humans and horses in the 1950s and 1960s. Lineage 1 was the only one circulating in Europe until 2004, when type 2 lineage was identified in Hungary, after which it spread into other countries. Since 2010, a geographical expansion with marked seasonality has been observed. Currently, lineage 2 is responsible for the majority of human cases in Europe [3-5]. From 2010 to 2019, locally acquired human cases were detected every year in the European Union/European Economic Area (EU/EAA), with two large outbreaks in 2010 (391 cases, mainly affecting Greece with 262 cases) and 2018 (1,615 cases throughout the EU/EAA). The notification rate for locally acquired WNV infections in the EU/EEA was almost eight times higher in 2018 compared with 2017 [6]. During the 2020 season, EU/EEA and EU-neighbouring countries reported 336 locally acquired human cases of WNV infection [7].

In Spain, WNV was first documented in the early 1980s, in a retrospective study of sera from the population of Catalonia [8]. Since 2010, equid outbreaks and wild bird cases have been reported in Andalusia, Extremadura, Castilla-La Mancha, Castilla y León and Catalonia. The first human case of WNND infection was retrospectively detected in a person who stayed in Badajoz in 2004 [9]. In 2010, two human cases were detected in Andalusia. In 2016, a considerable increase in equid outbreaks was observed (73 outbreaks), and three human cases of WNND were identified in people who had visited municipalities of Seville. From 2017 to 2020, WNV activity was low, with few equid outbreaks (13 in 2017, nine in 2018 and, in 2019, four in Andalucía, one in Extremadura and one in Catalonia) and none in humans. Before 2020, nearly all WNV strains detected in Spain were lineage 1, with the exception of one strain of lineage 2 that was detected in a goshawk in Lleida in 2017, as well as an exceptional finding of a new lineage in mosquitoes in 2006 [10,11].

### Outbreak detection

**Figure 1:**
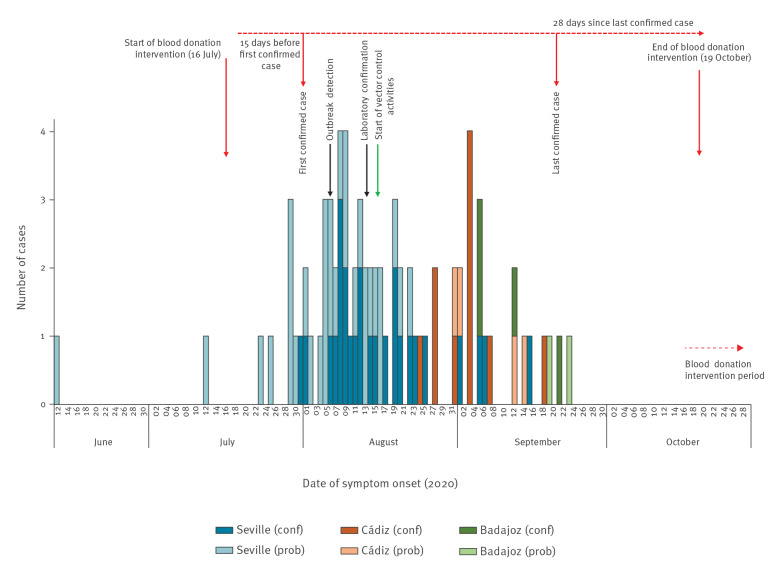
Epidemic curve of confirmed and probable human cases of West Nile virus infection, by province, Spain, as at 30 November 2020 (n=77)

In August 2020, a series of five human cases with WNND presenting as lymphocytic meningoencephalitis were identified in two neighbouring municipalities of Seville province, Andalusia. Simultaneously, four WNV outbreaks were reported in equid holdings in Andalusia. The first human cases were laboratory confirmed on 13 August (Figure 1). As this was an unprecedented situation, the National Epidemiological Surveillance Network and the Coordinating Centre of Health Alerts and Emergencies (CCAES) were informed and regional authorities implemented control measures in their territories. One month later, on 11 September, human WNND cases were also identified in Cádiz, and then in Badajoz on 22 September.

conf: confirmed; prob: probable.

Source: National Epidemiological Surveillance Network.

Given the large number of cases notified in the 2020 season, we aimed to analyse the epidemiological data, to document the measures taken and to perform a risk assessment to obtain recommendations for further public health measures.

## Methods

### Case definition

In accordance with the national guidelines put in place in 2020 and the EU case definition, a human case of WNND was suspected when a person who lived in or visited a risk area, or was bitten by mosquitoes, presented with at least one of the following signs or symptoms: encephalitis, meningitis, acute flaccid paralysis, Guillain–Barré syndrome or fever > 38.5 °C. To confirm the case, at least one of the following laboratory criteria was required: virus culture, nucleic acid detection in a clinical sample, IgM detection in cerebrospinal fluid (CSF) or detection of IgM and IgG on sera with confirmation by neutralisation assay [12,13]. Cases were classified as probable if specific IgM response was detected in a serum sample.

To confirm the presence of WNV in human urine samples, we used a specific real-time PCR for WNV [14] and we obtained sequences using two different reverse-transcriptase -nested-PCRs generic for flaviviruses [15,16].

### Surveillance in humans

Regional and national surveillance systems served as data sources to detect the outbreak. At the regional level, human meningoencephalitis cases are under surveillance in several regions in Spain, including in Andalusia, where the first cases in this outbreak were detected. A detection of a single case of human WNND is considered a public health alert, and therefore requires an urgent communication to both regional and national levels. The surveillance network at the national level includes the CCAES at the Ministry of Health, the National Centre of Epidemiology (NCE) and the National Centre for Microbiology (NCM). The CCAES coordinates all actions taken in the different regions and with other stakeholders, such as the Ministry of Agriculture, Fisheries and Food; the Scientific Committee for Blood Transfusion Security; and the Superior Council of Scientific Investigations.

The NCE manages and hosts the database and communication platform (SiViEs) of the national surveillance system [12]. Since 2010, all regions communicate epidemiological data using an agreed upon epidemiological protocol that contains diagnostic criteria; public health measures, e.g. seasonal active surveillance of meningoencephalitis cases in regions considered at risk; and options for response activities in the case of an outbreak. The protocol also contains a survey that includes sociodemographic (age, sex, place of residence), exposure (place of infection, risk factors) and clinical variables (date of onset, clinical criteria, hospitalisation, death); case classification (confirmed or probable); and laboratory data (date, criteria, specimen). Data should be provided as soon as they are available, and should be updated according to the evolution of a case. Initial microbiological investigations in relation to a public health alert should be shared with the NCM. Confirmation of cases and further microbiological investigations are performed at the NCM. The NCM also gives technical support to any region, upon request.

### Surveillance in equids, birds and mosquitoes

On the animal side, a national plan of WNV surveillance of horses and birds was established in 2007, and every year it is enhanced during the mosquito activity season between April and November [17,18]. The Ministry of Agriculture, Fisheries and Food records real-time information about WNV disease foci in equid holdings and cases in birds. Surveillance in equines is based on the investigation of animals showing clinical signs compatible with WNV disease. This is complemented by passive surveillance through sampling of sentinel animals located in areas considered at higher risk for WNV occurrence. These serum samples are taken monthly for enzyme-linked immunosorbent assay (ELISA) total antibodies test. In the case of positive results, serum samples are sent to the national reference laboratory (NRL) in Algete, Madrid where a serum neutralisation test (SNT) and ELISA for detection of anti-IgM antibodies is performed.

Passive surveillance in wild birds and birds at recovery centres is performed to detect clinical signs or abnormal mortality. In poultry, active surveillance is also performed in sentinel holdings on the same dates and in areas where positive birds are detected, which can be useful to detect seroconversion [18].

The vector-borne diseases response plan in Spain currently comprises the surveillance of *Aedes* sp. mosquitoes, but not *Culex*. Therefore, information about *Culex* mosquitoes comes from scientific research groups (i.e. Doñana Biological Reserve) in collaboration with the NCM and the NCE.

### Risk assessment

Risk assessment was performed by CCAES using the European Centre for Disease Prevention and Control (ECDC) methodology, taking into account the risk of transmission and the impact of the disease [19]. For the assessment of the risk of transmission, all data from humans, equids, birds and mosquitoes were taken into account. The impact of the disease was assessed considering the natural history of the disease and hospitalisation data registered during the outbreak, as well as the availability of diagnostic capacity and effective treatments [19].

### Ethical statement

This study involves the use of patient medical data from RENAVE, which is the official source for public health surveillance activities. RENAVE follows the mandate of Spanish and international legislation and is hosted by public institutions. Individual informed consent is not required for data to be included in RENAVE, and all data are pseudonymised, meeting all considerations regarding personal data protection. As this work is in line with alert, response and surveillance activities, no explicit ethics assessment was required.

## Results

### Human cases of West Nile virus infection

As at 30 November 2020, 77 human cases of WNV infection were reported, 40 (52%) of which were confirmed (Table 1). The age of cases ranged from 4 to 88 years old (median: 65; interquartile range (IQR): 45–76) and 46 cases (59.7%) were males. The dates of symptom onset ranged from 12 June to 23 September 2020 (Figure 1). Seventy-two cases (93.5%) presented neurological symptoms and five cases only reported fever (Table 1). Among the WNND cases, 40 (55.5%) had meningoencephalitis, 12 (16.7%) had encephalitis and 20 (27.8%) had meningitis. Of the 73 cases (94.8%) admitted to hospital, seven died and two remained hospitalised because of severe neurological sequelae. The distribution of the infections, by province (equivalent to the nomenclature of territorial units for statistics (NUTS) 3 [20]), and main epidemiological characteristics are also shown in Table 1. Laboratory diagnosis was established in five of the confirmed cases by virus nucleic acid detection in urine and by IgM positivity in cerebrospinal fluid in the 35 others. All 37 probable cases were diagnosed by a positive IgM result in serological tests in blood or serum.

**Table 1:** Distribution of human West Nile virus infection cases by epidemiological and clinical characteristics, Spain, 2020 (n = 77)

Epidemiological and clinical characteristics	Confirmed	Probable	Total
**Province (equivalent to NUTS-3)**			
Seville	26	31	57
Cádiz	10	4	14
Badajoz	4	2	6
**Month of symptom onset**			
June	0	1	1
July	1	7	8
August	25	24	49
September	14	5	19
**Clinical presentation**			
Neurological symptoms	40	32	72
Fever	0	5	5
**Hospital admission**			
Yes	39	34	73
No	1	3	4
**Outcome**			
Discharged or not admitted	32	35	67
Still hospitalised^a^	2	1	3
Death	6	1	7
**Total**	**40**	**37**	**77**

NUTS: Nomenclature of territorial units for statistics [20].

^a^ As at 30 November 2020.

**Figure 2:**
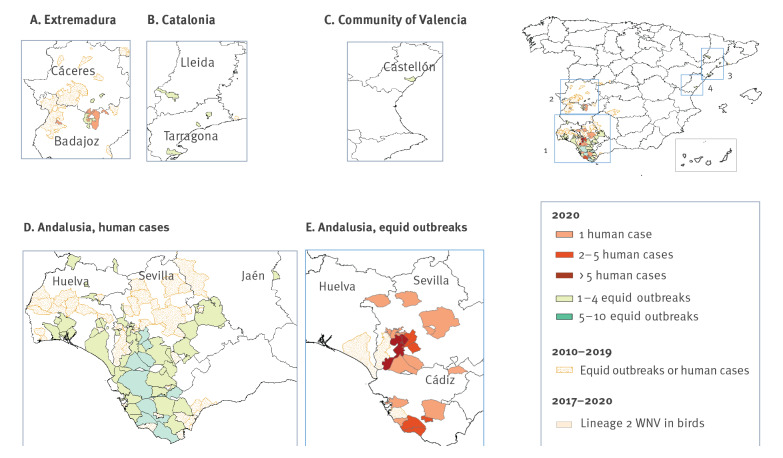
Equid outbreaks and human cases of West Nile virus (WNV) infection by region and WNV lineage 2 detection in wild birds, Spain 2010–2020

Source: National Epidemiological Surveillance Network and Ministry of Agriculture.

**Figure 3:**
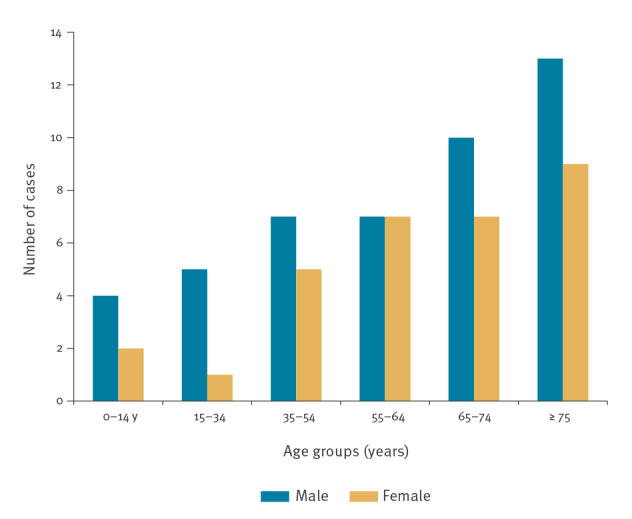
Distribution of confirmed and probable human West Nile virus cases, by age and sex, Spain, 2020 (n = 77)

Source: National Epidemiological Surveillance Network.

Among cases from Seville, the most affected province, 38 of 57 resided in two municipalities: Coria del Río or La Puebla del Río (Figure 2). Case ages ranged from 4 to 88 years old (median: 65; IQR: 45–76) and 34 (59.7%) were male (Figure 3).

### West Nile virus detections in equids

The first four equid foci were detected at the same time as the first human cases in four different livestock farms 30km and 100km, respectively, from La Puebla and Coria del Río, around the Guadalquivir river marshes. Over the full season, a total of 139 equid outbreaks have been reported (eight were in sentinel animals and the other 131 were identified through passive surveillance) in Seville (n = 58), Cádiz (n = 49), Huelva (n = 17), Badajoz (n = 5), Tarragona (n = 4), Lleida (n = 2), Cáceres (n = 2), Jaén (n = 1) and Castellón (n = 1) (Figure 3). In three provinces (Tarragona, Castellón and Jaén), WNV was detected for the first time this year [21].

### Circulating West Nile virus lineages

WNV sequencing was performed in four human cases and all belonged to lineage 1. No other flaviviruses were detected in these samples. Additionally, lineage 1 was identified in nine birds, 11 equines and 31 pools of *Culex perexiguus* and one pool of *Cx. pipiens* mosquitoes captured in Andalusia. During the summer, three goshawks were found dead and infected with WNV lineage 2 in Catalonia; one was found in Lleida and two in Tarragona, towns separated by more than 100 km [21].

### West Nile virus detections in *Culex* spp. and climatic variables

According to information from entomologists working in the field, vector activity in the 2020 WNV season was especially high in the areas of Puebla and Coria del Río, with abundant presence of *Cx. perexiguus* in the rice-growing areas. The maximum capture per night trap on the border of one of the affected villages in 2020 was 1,312 *Cx. perexiguus* and 11 *Cx. pipiens*, while in 2013 (the last year the trap was operated in the area) it was 12 *Cx. perexiguus* and 24 *Cx. pipiens* (data not shown). In relation to climatic variables, the spring was very rainy and precipitation in May was very high compared to what is usual in the area: 49.1 mm in 2020 compared with a mean of 15.2 mm (range 0–44.9 mm) between 2010 and 2019 [22].

### Risk assessment

The risk of transmission is considered moderate in extended areas in Spain where WNV has been detected in animals and/or human cases have already occurred, either during the 2020 season or in previous ones. The impact is considered high because of the severity and mortality usually associated with WNND and actually observed in the outbreak described. Additionally, even when the healthcare system is sufficiently prepared, there is no specific effective treatment available for WNND. In other territories, where WNV has never been detected in horses, birds or mosquitoes, the risk is considered lower, but ongoing extension of virus circulation into new territories is expected. During the winter months the risk is very low throughout Spain [23].

### Outbreak control measures

Addressing the outbreak through a multisectoral action plan and intensifying some activities in the areas of greatest risk was of crucial importance for proactive case detection and prevention and control of the disease. As the increase in cases was detected, the human, equine, bird and *Culex* mosquito surveillance activities were strengthened in areas considered at risk. Further environmental investigations were performed at the local level by regional public health authorities in collaboration with expert entomologists deployed in the field. Entomologists identified habitats with an abundance of competent vectors and recommended adequate vector control strategies.

Performing PCR tests on human urine samples was included in the national protocols to increase the diagnostic yield. Healthcare providers were alerted so that clinicians could increase their level of suspicion and consider WNV infection as a differential diagnosis. The populations living in the most affected areas were informed about the outbreak through mass media and were advised to use protection against mosquito bites. In the areas where control measures were implemented, they were effective in decreasing transmission. Nevertheless, human WNV infection cases were still detected in other areas, far from the initial outbreak area (Figure 1).

Actions were also taken to guarantee the safety of substances of human origin, i.e. blood products, organs, cells and tissues, in accordance with the Ministry of Health regulations, the National Transplant Organisation and the European Commission directive 2014/110/UE [24,25]. A retrospective screening of all donations in the 15 days before the onset of symptoms of the first confirmed human case was implemented in the affected areas (Sevilla, Cádiz and Badajoz). Potential donors who had lived in or visited the affected areas within the 28 days before donation were either deferred from donation or screened for WNV by RT-PCR. In this screening, four positive asymptomatic WNV cases were identified. Additionally, a stored sample of plasma was positive for WNV. Red blood cells from the same donor had been already transfused to a recipient, who experienced asymptomatic seroconversion after the transfusion (data not shown).

Timely communications were issued through the EU Early Warning Response System to inform the European Commission and EU countries, and cases were notified to the European Surveillance System operated by ECDC from the beginning of the outbreak and throughout the follow-up.

Additional recommendations were given to regional authorities. They were advised to implement active surveillance of lymphocytic meningoencephalitis in people residing in or visiting risk areas, including testing for WNV. Moreover, in current and future seasons vector control activities, mainly against the larval stages, should be strengthened locally to reduce the risk of WNV transmission. Conducting virological studies in order to determine eventual changes of the circulating WNV strains associated with increased virulence or transmissibility was also highly recommended. Furthermore, a review and update of the national guidelines for surveillance and rapid risk assessments were performed [23].

Actions taken regarding animals were aimed at the early detection of WNV. Equid outbreaks were communicated to ECDC and any positive WNV test in equines or birds was immediately reported to the public health authorities. Epidemiological update reports were shared with the regional authorities and other stakeholders, as well as veterinary and hunting associations. Efforts were made to raise awareness of the potential for WNV circulation, stressing the importance of reporting any suspicion of disease in equines, as well as abnormal signs or mortality in wild birds. In the high-risk areas, disinsection of holdings and voluntary vaccination of equines were recommended. In accordance with the Terrestrial Animal Health Code of the World Organisation for Animal Health, no restrictions on the movement of equids were imposed in any case, as these animals did not represent a risk for the transmission of the disease to other animals, nor to humans.

Actions to control the vector population were developed first around the area of the Coria-Puebla del Río outbreak and later for the province of Cádiz, as they have different environmental characteristics. Vector control actions were planned in two phases: (i) the shock phase, consisting of responding urgently to eliminate the majority of the adult mosquito population that could be associated with the outbreak in urban centres and peri-urban areas of the municipalities where confirmed cases were detected and (ii) the larvicide phase, which involved developing new actions to control the vector and its larvae, with a greater territorial expansion than the peri-urban area (prioritising municipalities with confirmed human cases) and monitoring progress through weekly meetings with the companies responsible for the application until the end of the vector activity period.

## Discussion

The massive increase in the incidence of WNND in the summer of 2020 was unprecedented in Spain. According to information from entomologists working in the field, this season vector activity was especially high in the rice-growing areas. In urban areas, *Cx- pipiens* probably took advantage of scuppers and other structures with water to reproduce because of a lack of actions to prevent their reproduction. It is likely that the coronavirus disease pandemic influenced the decline in vector control activities, as many resources needed to be redirected to pandemic response. The large geographical distribution of animal and human cases suggests that a common factor may be playing a role, either the vector density or a change in the transmissibility or virulence of the circulating WNV strain. The sequence of WNV obtained in this outbreak from four human samples in a short fragment of the conserved NS5 gene showed a few differences from the strains detected previously in Spain from birds, horses and mosquitoes (FJ766331, JF719069 and JF707789, respectively [26]). More information about the WNV genome in samples from this season would help to increase understanding of the current outbreak. The geographical position of the Iberian Peninsula, in the middle of bird migratory routes, might favour the (re)introduction of new WNV strains.

The fact that many human WNV infections are asymptomatic or mild—together with the existence of a number of self-limited meningitis cases [27], especially in young and immunocompetent people—might favour an underestimation of WNND human cases [28]. This could partially explain the low frequency and/or absence of human cases in previous seasons. However, there is a lack of clinical suspicion, probably related to WNND being considered as a rare, non-endemic disease. Even though laboratory tests are performed when WNV infection is suspected, diagnosis has its difficulties; therefore, a combination of molecular and serological assays are recommend for the complete diagnosis of WNV infection.

Improved diagnosis, surveillance and research around WNV could also contribute to a better knowledge of other pathogens such as Usutu virus (USUV), which shares ecological characteristics and clinical features with human WNV infections. USUV genome has been found in mosquitoes [29,30] and birds [31] in Spain, and specific antibodies have been reported in birds, horses and several mammals at a zoo [32-35]. The co-circulation of both viruses, which has been demonstrated in Italy [36], could also happen in Spain, although no evidence of USUV circulation was found in the mosquitoes investigated during the WNV outbreak.

At the end of 2020, the national plan for WNV surveillance in horses and birds was updated and added entomological surveillance with a double objective: to identify mosquito species and their abundance, and to detect viral circulation, as mosquitoes are good indicators of viral circulation in an area because of their limited movements [18]. The surveillance of *Culex* mosquitoes will contribute to a better understanding of the circulation of WNV—as well as other pathogens—in our territory.

## Conclusions

The 2020 WNV outbreak in Spain, detected by surveillance systems, highlights the importance of maintaining such systems to detect eventual increases of virus circulation. The unprecedented increase of WNND cases, along with previously known circulation of WNV and the existence of undiagnosed cases of viral meningoencephalitis, was challenging and required us to improve our capacity to detect human cases of WNND and to better prepare for next seasons, including raising awareness among clinicians and developing adequate laboratory diagnosis, especially in risk areas and for WNND cases. Strengthening active surveillance in territories where WNV has never been detected in horses, birds or mosquitoes would improve knowledge about virus circulation and thus the risk of virus transmission to humans. Going forward, maintaining vector control activities and updating the vector-borne diseases response plan in Spain is needed.

## References

[1] Patel H Sander B Nelder MP. Long-term sequelae of West Nile virus-related illness: a systematic review. Lancet Infect Dis. 2015;15(8):951-9. 26163373 10.1016/S1473-3099(15)00134-6

[2] Petersen LR Brault AC Nasci RS. West Nile Virus: Review of the Literature. JAMA. 2013;310(3):308-15. 23860989 10.1001/jama.2013.8042 PMC4563989

[3] Bakonyi T Ferenczi E Erdélyi K Kutasi O Csörgő T Seidel B Explosive spread of a neuroinvasive lineage 2 West Nile virus in Central Europe, 2008/2009. Vet Microbiol. 2013;165(1-2):61-70. 23570864 10.1016/j.vetmic.2013.03.005

[4] Papa A Bakonyi T Xanthopoulou K Vázquez A Tenorio A Nowotny N. Genetic characterization of West Nile virus lineage 2, Greece, 2010. Emerg Infect Dis. 2011;17(5):920-2. 21529413 10.3201/eid1705.101759 PMC3321789

[5] Chancey C Grinev A Volkova E Rios M. The Global Ecology and Epidemiology of West Nile Virus. BioMed Res Int. 2015;2015:376230. 25866777 10.1155/2015/376230 PMC4383390

[6] European Centre for Disease Prevention and Control (ECDC) and European Food Safety Authority. (EFSA). The European Union One Health 2019 Zoonoses Report. Stockholm: ECDC; 2021. Available from: https://www.ecdc.europa.eu/sites/default/files/documents/zoonoses-EU-one-health-2019-report.pdf 10.2903/j.efsa.2021.6406 PMC7913300 33680134

[7] European Centre for Disease Prevention and Control (ECDC). Epidemiological update: West Nile virus transmission season in Europe, 2020. Stockholm: ECDC; 2021. Available from: https://www.ecdc.europa.eu/en/news-events/epidemiological-update-west-nile-virus-transmission-season-europe-2020

[8] Lozano A Filipe AR. Anticuerpos frente a virus West Nile y otros virus transmitidos por artropodos en la poblacion del Delta Del Ebro. Rev Esp Salud Publica. 1998;72(3):245-50. 10.1590/S1135-57271998000300009 9810831

[9] Kaptoul D Viladrich PF Domingo C Niubó J Martínez-Yélamos S De Ory F West Nile virus in Spain: report of the first diagnosed case (in Spain) in a human with aseptic meningitis. Scand J Infect Dis. 2007;39(1):70-1. 10.1080/00365540600740553 17366016

[10] Busquets N Laranjo-González M Soler M Nicolás O Rivas R Talavera S Detection of West Nile virus lineage 2 in North-Eastern Spain (Catalonia). Transbound Emerg Dis. 2019;66(2):617-21. 30506625 10.1111/tbed.13086 PMC7380044

[11] Vazquez A Sanchez-Seco MP Ruiz S Molero F Hernandez L Moreno J Putative new lineage of west nile virus, Spain. Emerg Infect Dis. 2010;16(3):549-52. 20202444 10.3201/eid1603.091033 PMC3322021

[12] Instituto de Salud Carlos III (ISCIII). Fiebre del Nilo occidental. [West Nile Virus]. Madrid: ISCIII. [Accessed: 10 May 2021]. Spanish. Available from: https://www.isciii.es/QueHacemos/Servicios/VigilanciaSaludPublicaRENAVE/EnfermedadesTransmisibles/Documents/PROTOCOLOS/Protocolo%20vigilancia%20fiebre%20Nilo%20occidental_RENAVE.pdf

[13] European Commission. Commission implementing decision (EU) 2018/945 of 22 June 2018 on the communicable diseases and related special health issues to be covered by epidemiological surveillance as well as relevant case definitions. Official Journal of the European Union. Luxembourg: Publications Office of the European Union. 6.7.2018: L170/1. Available from: https://eur-lex.europa.eu/eli/dec_impl/2018/945/oj

[14] Vázquez A Herrero L Negredo A Hernández L Sánchez-Seco M Tenorio A. Real time PCR assay for detection of all known lineages of West Nile virus. J Virol Methods. 2016;236:266-70. 27481597 10.1016/j.jviromet.2016.07.026

[15] Sánchez-Seco M Rosario D Domingo C Hernández LKV Guzmán M Tonorio A Generic RT-nested-PCR for detection of flaviviruses using degenerated primers and internal control followed by sequencing for specific identification. J Virol Methods. 2005;126(1-2):101-9. 15847925 10.1016/j.jviromet.2005.01.025

[16] Vázquez A Sánchez-Seco M Palacios G Molero F Reyes N. Novel flaviviruses detected in different species of mosquitoes in Spain. Vector Borne Zoonotic Dis. 2012;12(3):223-9. 22022811 10.1089/vbz.2011.0687 PMC3300060

[17] Ministry of Agriculture. Plan de vigilancia de la encefalitis del Oeste del Nilo (West Nile) en España. [Surveillance plan for West Nile encephalitis in Spain]. Madrid: Ministry of Agriculture, Fisheries and Food; 2012. Spanish.

[18] Ministry of Agriculture. Fisheries and Food. Programa de Vigilancia de fiebre del Nilo occidental 2021. [West Nile Fever Surveillance Program 2021]. Madrid: Ministry of Agriculture, Fisheries and Food; 2020. Spanish. Available from: https://www.mapa.gob.es/es/ganaderia/temas/sanidad-animal-higiene-ganadera/programafiebredelnilooccidental2021_tcm30-437515.pdf

[19] European Centre for Disease Prevention and Control (ECDC). Operational tool on rapid risk assessment methodology - ECDC 2019. Stockholm: ECDC; 2019. Available from: https://www.ecdc.europa.eu/en/publications-data/operational-tool-rapid-risk-assessment-methodology-ecdc-2019

[20] European Commission. Regulation (EC) No 1059/2003 of the European Parliament and of the Council of 26 May 2003 on the establishment of a common classification of territorial units for statistics (NUTS). Official Journal of the European Union. Luxembourg: Publications Office of the European Union. 21.06.2003:L 155. Available from: https://eur-lex.europa.eu/legal-content/EN/TXT/?uri=celex%3A32003R1059

[21] Ministry of Agriculture. Fisheries and Food. Actualización de la situación epidemiológica de la fiebre del Nilo occidental (West Nile fever). [Update on the epidemiological situation of West Nile Virus]. Madrid: Ministry of Agriculture, Fisheries and Food; 29 Jan 2021. Spanish. Available from: https://www.mapa.gob.es/es/ganaderia/temas/sanidad-animal-higiene-ganadera/informefno_2021-01-29_tcm30-435293.pdf

[22] Doñana Biological Reserve (EBD). Clima y Meteorología. [Climate and meteorology]. Huelva: EBD. [Accessed: 5 Apr 2021]. Spanish. Available from: http://icts.ebd.csic.es/es/datos-meteorologicos

[23] Spanish Ministry of Health. Meningoencefalitis por el virus del Nilo occidental en España (2^a^ actualización-cierre de temporada). [West Nile Meningoencephalitis in Spain (2nd update – en of season]. Madrid: Ministry of Health; 3 Dec 2020. Spanish. Available from: https://www.mscbs.gob.es/profesionales/saludPublica/ccayes/alertasActual/docs/20201203_ERR_Nilo_Occidental.pdf

[24] Scientific Committee for Transfusion Safety. Virus del Nilo Occidental. [West Nile Virus]. Madrid: Ministry of Health. [Accessed: 7 May 2021]. Spanish. Available from: https://www.mscbs.gob.es/profesionales/saludPublica/medicinaTransfusional/acuerdos/docs/Virus_Nilo_Occidental.pdf

[25] Organización Nacional de Trasplantes (ONT). [National Transplant Organisation]. Documento de Consenso del Grupo de Estudio de la Infección en el Trasplante (GESITRA) perteneciente a la Sociedad Española de Enfermedades Infecciosas y Microbiología Clínica (SEIMC) y la Organización Nacional de Trasplantes (ONT) sobre los Criterios de Selección del Donante de Órganos Sólidos en Relación a las Enfermedades Infecciosas.[ Consensus Document of the Transplant Infection Study Group (GESITRA) belonging to the Spanish Society of Infectious Diseases and Clinical Microbiology (SEIMC) and the National Transplant Organization (ONT) on the Selection Criteria for Solid Organ Donors in Relationship to Infectious Diseases]. Madrid: ONT; 2019. Spanish. Available from: http://www.ont.es/infesp/DocumentosDeConsenso/GESITRA_ONT_SEIMC_WEB_mayo2020.pdf

[26] Sotelo E Fernández-Pinero J Llorente F Vázquez A Moreno A Agüero M Phylogenetic relationships of Western Mediterranean West Nile virus strains (1996-2010) using full-length genome sequences: single or multiple introductions. J Gen Virol. 2011;92(11):2512-22. 21775579 10.1099/vir.0.033829-0

[27] McGill F Griffiths MJ Bonnett LJ Geretti AM Michael BD Beeching NJ Incidence, aetiology, and sequelae of viral meningitis in UK adults: a multicentre prospective observational cohort study. Lancet Infect Dis. 2018;18(9):992-1003. 30153934 10.1016/S1473-3099(18)30245-7 PMC6105576

[28] Gibbons CL Mangen M-JJ Plass D Havelaar AH Brooke RJ Kramarz P Measuring underreporting and under-ascertainment in infectious disease datasets: a comparison of methods. BMC Public Health. 2014;14(1):1-17. 24517715 10.1186/1471-2458-14-147 PMC4015559

[29] Vázquez A Ruiz S Herrero L Moreno J Molero F Magallanes A West Nile and Usutu viruses in mosquitoes in Spain, 2008-2009. Am J Trop Med Hyg. 2011;85(1):178-81. 21734145 10.4269/ajtmh.2011.11-0042 PMC3122364

[30] Busquets N Alba A Allepuz A Aranda C Nuñez JI. Usutu Virus Sequences in Culex pipiens (Diptera: Culicidae), Spain. Emerg Infect Dis. 2008;14(5):861-3. 18439389 10.3201/eid1405.071577 PMC2600269

[31] Höfle U Gamino V de Mera I Mangold A Ortíz J de la Fuente J. Usutu virus in migratory song thrushes, Spain. Emerg Infect Dis. 2013;19(7):1173-5. 23764143 10.3201/eid1907.130199 PMC3713991

[32] Llorente F Pérez-Ramírez E Fernández-Pinero J Soriguer R Figuerola J Jiménez-Clavero MÁ. Flaviviruses in Game Birds, Southern Spain, 2011-2012. Emerg Infect Dis. 2013;19(6):1023-5. 23735195 10.3201/eid1906.130122 PMC3713840

[33] Bravo-Barriga D Aguilera-Sepúlveda P Guerrero-Carvajal F Llorente F Reina D Pérez-Martín JE West Nile and Usutu virus infections in wild birds admitted to rehabilitation centres in Extremadura, western Spain, 2017-2019. Vet Microbiol. 2021;255:109020. 33677369 10.1016/j.vetmic.2021.109020

[34] Vanhomwegen J Beck C Desprès P Figuerola A García R. Circulation of Zoonotic Arboviruses in Equine Populations of Mallorca Island (Spain). Vector Borne Zoonotic Dis. 2017;17(5):340-6. 28346868 10.1089/vbz.2016.2042

[35] Caballero-Gómez J Cano-Terriza D Lecollinet S Carbonell M Martínez-Valverde R. Evidence of exposure to zoonotic flaviviruses in zoo mammals in Spain and their potential role as sentinel species. Vet Microbiol. 2020;247:108763. 32768215 10.1016/j.vetmic.2020.108763

[36] Grottola A Marcacci M Tagliazucchi S Gennari W Di Gennaro A Orsini M Usutu virus infections in humans: a retrospective analysis in the municipality of Modena, Italy. Clin Microbiol Infect. 2017;23(1):33-7. 27677699 10.1016/j.cmi.2016.09.019

